# Ultraviolet-C Light-emitting Device Against Microorganisms in Beauty Salons

**DOI:** 10.20411/pai.v7i1.497

**Published:** 2022-06-16

**Authors:** Margarete Teresa Gottardo de Almeida, Bianca Gottardo de Almeida, João Paulo Zen Siqueira, Gabriela Byzynski Soares, Vinicius Sigari Morais, Fátima Maria Mitsue Yasuoka, Filippo Ghiglieno

**Affiliations:** 1 Faculdade de Medicina de São José do Rio Preto (FAMERP), São José do Rio Preto, Brazil; 2 Universidade Júlio de Mesquita Filho (UNESP), campus of São José do Rio Preto (Ibilce), São José do Rio Preto, Brazil; 3 Nanochemtech Solutions, São José do Rio Preto, Brazil; 4 BR-Labs Tecnologia Óptica e Fotônica Ltda., São Carlos, Brazil; 5 Universidade Federal de São Carlos (UFSCar) – Laboratório de Óptica, Laser e Fotônica (OLAF), São Carlos, Brazil.

**Keywords:** Ultraviolet light, Pathogen Transmission, Decontamination, Occupational Safety, Primary Prevention, Beauty Culture.

## Abstract

**Background.:**

Ultraviolet light in the UV-C band is also known as germicidal radiation, and it is widely used for decontamination and disinfection of environments, water, and food. The ultraviolet source transfers electromagnetic energy from a mercury arc lamp to an organism's genetic material. When UV radiation penetrates the cell wall of an organism, it destroys the cell's ability to reproduce, through a physical and not chemical process. Thus, the objective of this study was to evaluate the antimicrobial potential of a new UV-C generating device (Asepsis) against clinically important microorganisms that may be present in beauty centers.

**Methods.:**

We present here a set of tests performed on tools easy to find in beauty salons (hair-brushes, nail pliers, makeup brushes, and, due to the recent COVID-19 pandemic, face mask samples). They were individually contaminated with bacteria (*Pseudomonas aeruginosa, Staphylococcus aureus*), fungi (*Microsporum canis, Trichophyton rubrum, Candida albicans, Malassezia furfur*), and the Chikungunya virus. Different times of exposure were evaluated (1, 3, and 5 minutes).

**Results.:**

There was notable reduction in the microbial load in every test, in comparison with control groups. Best results were observed on face mask samples, while the makeup brush showed less reduction, even with longer periods of exposure.

**Conclusions.:**

Beauty salons present a risk of infections due to microbial exposure. The device tested can efficiently inactivate, in a short time, microorganisms contaminating most tools found in this setting. The device also showed promising results against enveloped virus.

## INTRODUCTION

In modern times, the demand for beauty care and cosmetic treatments is rapidly increasing. Consequently, the beauty salon industry has been growing at a high rate in recent years. In 2020, even with the COVID-19 pandemic, the global market for beauty salons and spas was estimated at US$139 billion, and projections are optimistic [1]. According to the Brazilian Association of the Personal Hygiene, Perfumery, and Cosmetics Industry (ABIHPEC), there are currently about 5.5 million job opportunities in this sector in Brazil, including people already working and job vacancies. In the USA and Europe, regarding only hairdressers and beauticians, there are currently almost 1.2 million and over 1.7 million people employed, respectively [2, 3].

People seek beauty salons and barber shops for professional cosmetic treatments, such as hairdressing, skin and nail care, makeup, and others. Because of these activities, employees and customers are subjected to a series of hazards, including chemical exposure and microbial contamination [4].

Infection risk in beauty centers varies depending on the activity and level of hygiene procedures, such as use of personal protective equipment (PPE), sanitization, sterilization, and disinfection of instruments and devices. However, due to the ubiquity of microorganisms and high probability of transmission, the instruments could act as fomites even if the site is not apparently contaminated [4,5]. Fomites are objects contaminated by microorganisms that may act as route of transmission of infections [6].

These microbes can take advantage of a weakened immune system of the host or breached integumentary barriers and establish an infection. Brushes, combs, scissors, files, nail pliers, tweezers, among others, could harbour potentially pathogenic organisms and facilitate the transmission of infectious diseases [7].

Microorganisms, on fomites or in the environment, can remain viable for hours, or even days, depending on the fomite material, microorganism type, and indoor environmental characteristics [8]. The most common organisms associated with beauty salons are bacteria, such as *Streptococcus, Staphylococcus, Enterococcus, Micrococcus, Enterobacter*, and Mycobacteria; *Candida* and dermatophytes fungi; and viruses, for example, HBV and HCV [9–13].

Routine disinfection practices may not be sufficient to eliminate contamination of surfaces and fomites [14]. For example, cleaning chemicals, such as detergents, may displace and solubilize the contamination without inactivating microorganisms [15]. Proper decontamination protocols should be fast, efficient, and simple. Ultraviolet (UV) light devices can be powerful tools, alone or in combination with other methods, because of their broad spectrum of activity, being widely used for water and food disinfection [16]. The UV radiation, which can be differentiated by wavelength as A, B, or C, affects the DNA, inducing damaging mutations [17]. Several factors can influence the activity of UV light, for example, the amount of irradiance, distance, angle at which the UV strikes, and type of radiation [18]. Ultraviolet C (UV-C) radiation has the shortest wavelength (100–280 nm) and the highest energy (4.42–12.42 eV) [19].

The objective of this study was to evaluate the antimicrobial potential of UV-C radiation generated by an innovative device against clinically important microorganisms that may be present in fomites of beauty centers.

## METHODS

**Figure 1.**
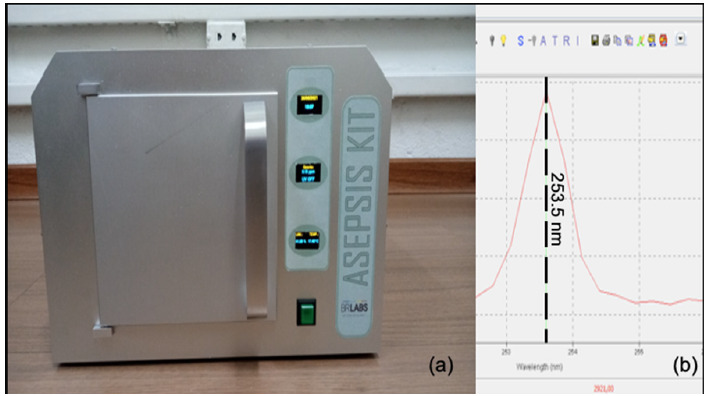
(A) Asepsis Kit, release 1.1 (courtesy of BR LABS Tecnologia Óptica e Fotônica Ltda); (B) lamp spectrum in the UV-C region.

A recently developed, state-of-the-art device called the Asepsis Kit (BR LABS^®^) (Figure 1A) is produced as an isolated case, optimized for UV light irradiation. The device is embedded with an electronic board that enables the user to easily program the exposure time to the radiation. Inside the case, temperature, humidity, and ozone concentration are monitored in real-time during the process.

The installed UV source was characterized on a USB4000 fiber optic spectrometer (Ocean Optics). The emission spectral lines are both in the UV and visible electromagnetic spectrum. The main peak in the UV-C region (100 nm – 280 nm) is centred at 253 nm (Figure 1B). The lamp parameters follow the technical rule n° 32/2021 for UV disinfecting purposes in the public environment and hospitals, recently published by the Brazilian National Health Surveillance Agency (ANVISA) [20].

The procedure consisted of contaminating hairbrushes, nail pliers, makeup brushes, and face mask samples with several pathogenic microorganisms, prior to exposure to the UV-C device. The device was used according to the manufacturer's instructions. Time of exposure was set to 1, 3, and 5 minutes. Clinical isolates (skin, nail, and scalp) from the culture collection of the Micro-biology Laboratory of São José do Rio Preto Medical School were used, including bacteria (*Pseudomonas aeruginosa* and *Staphylococcus aureus*), fungi (*Microsporum canis, Trichophyton rubrum, Candida albicans*, and *Malassezia furfur*) and an enveloped virus (Chikungunya virus). Ethical approval was not required for this study. Table 1 shows which instruments were contaminated with each microorganism.

**Table 1.** Microorganisms and tools tested.

Microorganism	Hairbrush	Nail plier	Makeup brush	Face mask
*Pseudomonas aeruginosa*				**X**
*Staphylococcus aureus*	**X**		**X**	**X**
*Microsporum canis*	**X**			
*Trichophyton rubrum*		**X**		
*Candida albicans*				**X**
*Malassezia furfur*	**X**		**X**	
*Chikungunya virus*				**X**

The instruments (brushes and pliers) and face mask samples (3 × 2 cm) were previously sterilized by ethylene oxide. Each instrument and mask sample were contaminated with 30 μL of a microbial inoculum, according to Table 1. The inocula were prepared at a concentration of 10^8^ CFU/mL, for bacteria, and 10^5^ CFU/mL, for fungi, and were applied with a micropipette on tips of the pliers and bristles of the brushes. After exposure to the UV light (1, 3, and 5 minutes), a sample was taken from each utensil with a disposable sterile loop and inoculated into the specific culture medium plate (Brain-heart infusion agar, for bacteria; Kimmig's agar with olive oil, for *Ma. furfur*; and Sabouraud-dextrose agar, for the other fungi). Plates were incubated according to each microorganism (24 hours at 35°C for bacteria; 48 hours at 30°C for *Candida* and *Malassezia*; and 7 days at 35°C, for *Trichophyton* and *Microsporum*). For each test, there was a control group using the same criteria (organism and time), but without the radiation exposure. Tests were performed in triplicate.

To assess the antiviral activity of the device against the Chikungunya virus, serial dilutions (10^−2^ to 10^−6^) of the cultured virus (titre of 2.2 × 10^7^ plaque forming units (PFU)/mL) were inoculated into the mask (100 µL of the viral solution and 900 µL of culture media). Vero cells adhered to 24-well microplates were prepared, and 200 µl of each dilution was inoculated, in duplicate. One hour after incubation, the viral inoculum was removed, and 1mL/well of the mixture (v/v) of 1% Carboxymethylcellulose (CMC) and Minimal Essential Medium (MEM) plus 1% fetal bovine serum (FBS) was added. Cells were incubated at 37°C, at 5% CO_2_ atmosphere. After 2 days, the medium was removed, and the living cells were fixed with 10% formaldehyde for 20 minutes at room temperature. After fixation, a 1% crystal violet solution for visualization and counting was added. The infectious titer was calculated, expressed by the number of plaque-forming units, according to the formula: PFU = number of plaques * reciprocal of dilution * reciprocal of the volume (mL).

## RESULTS

**Figure 2.**
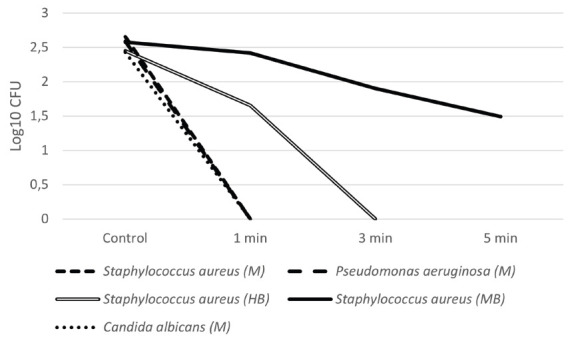
Average CFU count (Log_10_ CFU) of the microorganisms tested (*S. aureus, P. aeruginosa*, and *C. albicans*) after exposure to UV-C light according to the tested times (control, 1 min, 3 min, and 5 min). M: face mask, HB: hairbrush, MB: makeup brush.

**Figure 3.**
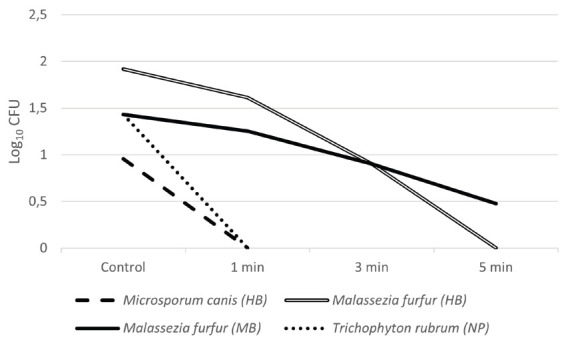
Average CFU count (Log_10_ CFU) of the microorganisms tested (*Mi. Canis, Ma. furfur*, and *T. rubrum*) after exposure to UV-C light according to the tested times (control, 1 min, 3 min, and 5 min). HB: hairbrush, MB: makeup brush, NP: nail pliers.

The results obtained in this study are summarized in Table 2. Regarding hairbrushes, inhibitory activity could be observed for all microorganisms tested, from 1 minute (Table 2). *S. aureus* was more susceptible to the treatment than *Ma. furfur*, with greater reduction observed after 3 minutes of exposure. For *Mi. Canis*, 1 minute was sufficient to prevent any growth (Figures 2 and 3).

**Table 2.** Average CFU count on the tests performed in this study, according to the time of exposure to the UV light.

	Hairbrush				Makeup brush				Nail plier				Face mask			
	C	1′	3′	5′	C	1′	3′	5′	C	1′	3′	5′	C	1′	3′	5′
*S. aureus*	283	45	0	0	377	260	80	31	-	-	-	-	450	0	0	0
*P. aeruginosa*	-	-	-	-		-	-	-	-	-	-	-	387	0	0	0
*C. albicans*	-	-	-	-		-	-	-	-	-	-	-	269	0	0	0
*Mi. Canis*	9	0	0	0	-	-	-	-	-	-	-	-	-	-	-	-
*Ma. furfur*	83	41	8	0	27	18	8	3	-	-	-	-	-	-	-	-
*T. rubrum*	-	-	-	-	-	-	-	-	27	0	0	0	-	-	-	-

C: control, -: not tested

Results of UV-C inhibitory activity against *T. rubrum* present in nail pliers were noteworthy (Table 2). Figure 3 show that this isolate was highly sensitive to UV-C, exhibiting no growth after exposure to the device.

Makeup brushes were contaminated with 2 microorganisms: *S. aureus* and *Ma. furfur*. Reduction was observed in every test in comparison to the control (Table 2). However, even the longest time of exposure (5 minutes) was not enough to eliminate all CFU in the samples (Table 2, Figures 2 and 3).

**Figure 4.**
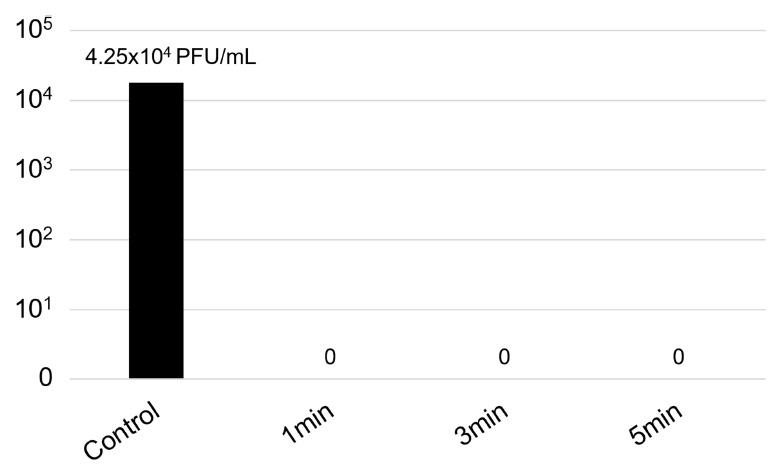
Viral titer (in PFU/mL) of the Chikungunya virus on face mask samples exposed to the UV-C device.

The activity of the device on face mask samples can be observed in Table 2 and Figures 2 and 4. Two bacteria (*P. aeruginosa* and *S. aureus*), a yeast (*C. albicans*), and the Chikungunya virus were tested. They were all inhibited after 1 minute of exposure.

## DISCUSSION

UV light technology has been widely used as a germicidal tool for water treatment and in the food industry. In addition, there are several commercial devices which have been developed to decontaminate surfaces and hospital rooms [21–23]. This study assessed the decontamination of beauty salon instruments by a UV-C light-emitting device, in order to reduce the risk of infections due to microbial exposure. The device was tested against bacteria, fungi, and virus, with reduction of microbial load at short times of exposure.

Several factors may influence the level of microbial contamination in the environment of a beauty salon. For example, high people flow, close employee/customer contact, the procedures performed, and the type of instruments used. The presence of microorganisms in these environments may lead to the transmission of important diseases, such as fungal nail infections, herpes, and skin rashes.

Disinfection of possible fomites is a crucial step to minimize the probability of transmission of infectious diseases. Fomites may carry potential pathogens from the hands of employees; hair, skin, or nails of the customers; or the environment (surfaces, air, water) [24]. For these reasons, disinfection and sterilization protocols should include methods with broad spectra of activity.

The device tested in this study can expose the main tools used in a beauty salon to UV-C light. The device is portable, user-friendly, time programmable, and minimizes shadowed areas. These features allow shorter times of exposure, increasing its effectiveness [25]. In addition, this technology can be beneficial over conventional chemical disinfection and thermal treatments. Chemical compounds may be toxic to the environment and thermal treatments may cause damage to certain materials [26, 27].

However, characteristics of the materials exposed to the device may alter the time needed for proper disinfection. For example, the bristles of a makeup brush are densely distributed, which may contribute to the retention of a larger number of microorganisms and obstruct exposure to the UV-C light. This is a possible explanation for why the makeup brushes presented less reduction of microbial load (Table 2). Complex shapes can also prevent UV penetration, decreasing the activity of the UV light [28]. An ‘*ad hoc*' designed support can overcome this issue. For example, a host holder inducing mechanical pressure on the hairbrushes can increase the exposed surface to the radiation, or a support softly stretching a mask can allow a deeper UV penetration.

The presence of organic matter on the instruments also may influence the efficacy of UV light decontamination. However, we were not able to assess a possible decrease of efficiency of the device in this study. To overcome this issue, manually cleaning the tools may remove excess organic matter and improve efficiency of the decontamination process [29].

With the recent COVID-19 pandemic, use of face masks increased, especially washable masks. If not effectively used and disinfected, they may constitute a risk of opportunistic infections. It has been demonstrated that UV light can reduce virus contamination of N95 respirators [30]. However, the level of disinfection may vary according to the material and the technology used. Here, we evaluated the efficacy of the UV device to disinfect reusable masks. All organisms tested were inhibited within 1 minute of exposure. We believe that the shape had a major influence on this result, with no shadowed areas and full exposure to the light.

In conclusion, according to the methodology tested, the device tested was able to inhibit many types of microorganisms after a short time of UV-C light exposure. The instruments that are being disinfected play an important role on the time of exposure needed. Further studies should be conducted to assess the optimal time of exposure to different material and shapes. In addition, the influence of organic matter and the combination of different disinfection techniques should be assessed. However, the results observed in this study show that this device may constitute a powerful aid to disinfection in the beauty salon setting.
